# Spontaneous Spinal Epidural Abscess Presenting in a Previously Healthy Young Adult Man

**DOI:** 10.1155/2013/872148

**Published:** 2013-08-13

**Authors:** Andrew M. McDonald, Jason L. Rollins

**Affiliations:** Department of Medical Education, Princeton Baptist Medical Center, 3201 4th Avenue S, Birmingham, AL 35222, USA

## Abstract

We report a case of spontaneous spinal epidural abscess (SEA) with initial chief complaint of shoulder pain and no appreciable neurologic symptoms. Since outcomes of SEA appear to be related to the degree of neurologic deficit at the time of intervention, we explore opportunities for earlier diagnosis.

## 1. Introduction

 Spinal epidural abscess (SEA) is an uncommon, though a well-described cause of acute neurologic deficit. Typically, an SEA develops in the setting of recent neurosurgical intervention, intravenous drug use, or known bacteremia from an identifiable source [1–4]. The most common presenting symptom is back or neck pain. Only slightly more than half of patients present with apparent neurologic dysfunction and a minority of patients demonstrate fever [1, 2]. Risk factors for the development of SEA include diabetes mellitus, immunosuppression, and recurrent skin infections or abscesses [5].

In this work, we present a case of a spontaneous SEA occurring in the absence of any of the aforementioned risk factors. 

## 2. Case Report

A 33-year-old man with no significant past medical history presented to the emergency department (ED) complaining of a 7-day history of constant, dull pain in his right shoulder with radiation to the ipsilateral neck and associated with slight decrease in sensation throughout the right upper extremity. With the exception of a pulse rate of 103 beats per minute, his vital signs were within normal limits. Examination of the neck and right shoulder was without abnormality. Plain radiographs of the right shoulder were obtained and were within normal limits. He was then given prescriptions for methylprednisolone, methocarbamol, and hydrocodone-acetaminophen and discharged home with a diagnosis of cervical radiculopathy.

 Two days later, he returned to the ED complaining of worsening pain in the right shoulder with radiation from the right arm to the neck with associated paresthesias in the right arm. Review of systems revealed interval development of nausea associated with occasional vomiting. Physical examination was notable for normal vital signs, focal tenderness of the right trapezius muscle with no range-of-motion deficits involving the right shoulder, and the absence of focal neurologic deficits. A point-of-care troponin measurement was undetectable. He was then provided a referral to undergo MRI as an outpatient and discharged home. 

**Figure 1:**
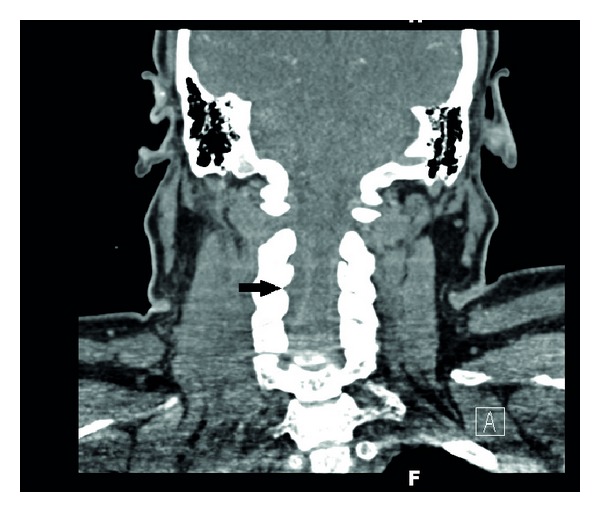
CT of the neck revealing a 3.7 by 1.4 cm low-density oval lesion (arrow) displacing the thecal sac and extending from the C2 to the C4 vertebrae.

Two days after his second presentation, the patient developed acute numbness of the right leg while waiting to be seen in an outpatient chiropractic clinic. He was instructed to immediately return to the ED, and no chiropractic manipulation was performed. However, by this time, the patient was unable to arise from a seated position and required transport to the ED by ambulance. Physical examination at the ED revealed diffuse muscle weakness and numbness distal to the neck, tachypnea, and mild diaphoresis. Vital signs were notable for pulse rate of 113 beats per minute, respiratory rate of 24, temperature of 99.5°F, pulse oximetry of 97% on room air, and blood pressure 165/98 mmHg. Noncontrast CT of the head was without abnormality, and erythrocyte sedimentation rate (ESR) was 105 seconds. The patient's respiratory status continued to worsen and was associated with an acute decrease in hemoglobin oxygen saturation by pulse oximetry. Arterial blood gas measurement revealed PO_2_ of 61 mmHg, and quantitative D-dimer was 3.64 mg/L (RR 0.19–0.49 mg/L); therefore, he was sent for CT angiogram which was negative for evidence of pulmonary thromboembolism but did reveal bilateral lower lobe consolidation. Shortly after the completion of the CT angiogram, he developed acute worsening of his respiratory distress requiring intubation. A previously ordered MRI was unable to be performed due to the unavailability of an MRI compatible ventilator. After intubation and stabilization, a contrast-enhanced CT of the cervical spine revealed a 3.7 by 1.4 cm low-density oval lesion displacing the thecal sac and extending from the C2 to the C4 vertebrae (Figure 1). 

Based on the imaging results, the patient was emergently taken to the operating room where he underwent laminectomy of C2–C5 for debridement of the epidural abscess, with the surgical wound left open for negative pressure wound vacuum therapy. He was also begun on intravenous vancomycin therapy. During postoperative day 1, due to the dense consolidation seen on chest CT, he underwent fiberoptic bronchoscopy with bronchial washings obtained for culture. The bronchial washings, as well as blood and abscess cultures, revealed methicillin-resistant *Staphylococcus aureus *(MRSA). An extensive evaluation, including transesophageal echocardiography, was undertaken in order to determine the source of MRSA infection in this young individual with no apparent risk factors, though no source was identified.

The remainder of the patient's hospital course was extremely complicated. By postoperative day 2, he developed acute respiratory distress syndrome with subsequent development of a right pneumothorax secondary to barotrauma. Two weeks after initial presentation, he was transitioned from vancomycin to linezolid given that his clinical status was not improving. On hospital day 16, he experienced cardiopulmonary arrest and was successfully resuscitated. He continued to experience bradycardia over the subsequent 2 weeks which was felt to be related to increased vagal tone. On hospital day 26, a percutaneous tracheostomy was placed and guarded weaning of sedation, paralytics, and ventilator support was begun. Blood cultures remained positive for MRSA until hospital day 28, after which they became repeatedly negative. He was eventually weaned completely from respiratory support and was deemed stable for discharge on hospital day 60 with a prescription for 4 weeks of oral doxycycline. At the time of discharge, he was able to freely move both of his upper extremities and exhibited slight movement in both of his lower extremities and was discharged to rehabilitation facility specializing in the treatment of spinal cord injuries.

## 3. Discussion

The development of a spontaneous SEA, one without an identifying cause or predisposing factor, is a rare occurrence, accounting for 0.88 cases per 100,000 [person years] by one estimate [6]. The rare nature of spontaneous SEA combined with only half of cases presenting with classic neurologic deficits leads to diagnostic difficulty [1, 2]. Despite this difficulty, the importance of early diagnosis cannot be overemphasized with Curry et al. reporting that only 38% of patients experienced improved neurologic function after treatment [2]. However, the neurologic status of an additional 33% remained unchanged from presentation, suggesting that an earlier diagnosis would have led to less long-term deficit. The primary goal of this case report was to identify key aspects of this patient's presentation and explore opportunities for earlier diagnosis.

Musculoskeletal complaints, such as shoulder and neck pain, are among the most common principal causes for seeking medical attention in the outpatient setting [7]. Initial evaluation should always include a thorough history and physical examination which is then utilized to choose which, if any, laboratory or imaging studies are indicated. For shoulder pain of any etiology, the American College of Radiology recommends traditional radiographs as the initial study [8]. MRI of the shoulder is recommended if radiographs are noncontributory in the setting of continued pain and nonspecific findings on history and physical examination, though the timing of this follow-up study is unspecified. 

When our patient presented to the ED for the second time, he exhibited continued shoulder pain despite medical treatment as well as interval development of paresthesias involving the right upper extremity. Outpatient MRI was scheduled due to lack of any focal neurologic findings on physical examination; however, additional physical exam maneuvers aimed at detecting cervical pathology may have been of benefit in this case and indicated a more urgent imaging study. A meta-analysis by Rubinstein et al. noted the Spurling maneuver, performed by rotating the head side to side while applying axial pressure to elicit paresthesias to be between 90% and 100% sensitive and 94% and 100% specific for cervical radiculopathy when combined with neck extension [9]. Additionally, the shoulder abduction test, performed by having the patient lift the affected arm and rest the hand upon the head while asking if symptoms improved, was greater than 75% specific [9]. While these physical examination maneuvers are useful for identifying the presence of underlying cervical pathology, they do not predict its severity. However, in the setting of worsening symptoms or a significantly prolonged ESR, the presence of these findings may suggest the need for urgent imaging evaluation to rule out severe cervical inflammatory pathology.

At the time of the patient's third presentations, the need for emergent CNS imaging was apparent. Despite the widespread utilization of MRI for the evaluation of neurologic deficits, contrast-enhanced CT scan receives equivalent ACR Appropriateness Criteria ratings for the diagnostic evaluation of an acute-onset focal neurologic deficit [10], though MRI remains the preferred imaging modality for the evaluation of symptoms consistent with acute, atraumatic myelopathy, particularly if an infectious etiology is suspected [11]. In the context of the presented case, an initial choice of CT imaging of the cervical spine would likely have resulted in fewer trips to the radiology suite, as this study could likely have been performed in rapid succession to the head or chest CT scans the patient underwent. Additionally, CT technology acquires images more rapidly than MRI, giving greater likelihood of obtaining a complete study in this unstable patient. 

The most appropriate treatment for SEA remains controversial due to lack of a published randomized trial. Classically, antimicrobial therapy with surgical decompression has been the treatment of choice [5], though one series of 57 cases suggested that antibiotic therapy alone, or in combination with percutaneous drainage, resulted in equivalent outcomes to surgical intervention [12]. Other authors suggest exercising caution when determining to forego surgery and that medical therapy alone is best reserved for patients with minimal neurologic deficits or who are poor surgical candidates [1, 4, 13]. With respect to our case, we recognize the immediate resolution of apparent mass effect, diagnostic confirmation, and procurement of fluid for culture as benefits of early surgical intervention.

## 4. Conclusions

 Spontaneous SEA is an uncommon condition whose initial presentation is often without appreciable neurologic deficit. Early diagnosis is paramount as the neurologic deficit does not always improve as a result of intervention. For patients presenting with shoulder pain and paresthesias, advanced physical exam techniques aimed at detecting cervical radiculopathy should be utilized to aid in determining if urgent imaging is indicated. The choice of imaging modality in an unstable patient should take into account the feasibility and speed of image acquisition in addition to image quality. The presence of a neurologic deficit is an indication for immediate surgical intervention in addition to antimicrobial therapy.
